# Case Report: Hypopituitarism Presenting With Nonconvulsive Status Epilepticus

**DOI:** 10.3389/fneur.2021.715885

**Published:** 2021-09-22

**Authors:** Huimin Li, Lina Xu, Fengbing Yang, Longbin Jia, Hongjiang Cheng, Wei Liu

**Affiliations:** Jincheng People's Hospital Affiliated to Shanxi Medical University, Jincheng, China

**Keywords:** nonconvulsive status epilepticus, hypopituitarism, hormone, seizure, case report

## Abstract

**Introduction:** Hypopituitarism is defined as one or more partial or complete pituitary hormone deficiencies. Nonconvulsive status epilepticus (NCSE) refers to a state of continuous or repetitive seizures without convulsions. In this paper, we review a case of an old female patient with hypopituitarism who presented with NCSE, which is rare in the clinic.

**Case Report:** This paper describes a 67-year-old female patient with hypopituitarism who presented as NCSE. She had surgical resection of pituitary tumor half a year before the seizures and did not get regular hormone replacement therapy. She presented general convulsive status epilepsy as the initial symptom and got sedation and antiepileptic drug in the emergency room. The seizure was terminated but the patient fell in coma in the following days. The patient had magnetic resonance imaging (MRI) and other inspects, and EEG showed epileptic discharges. Combining these clinical symptoms and examinations, we made the diagnosis of NCSE. Finally, she regained consciousness after the treatment with diazepam.

**Conclusion:** This case report and literature review investigated the possible mechanism of hypopituitarism presenting with NCSE.

## Introduction

Status epilepticus (SE) refers to a condition caused either by the failure of seizure termination or by the initiation of prolonged seizures. According to the classification of SE proposed by the International League Against Epilepsy (ILAE), NCSE is defined as SE without prominent motor symptoms, including NCSE with coma and NCSE without coma (1). The role of hormones in the pathophysiology of epilepsy has been gradually recognized (2, 3). In this paper, we report a patient with hypopituitarism presenting with NCSE.

## Case Report

**Figure 1:**
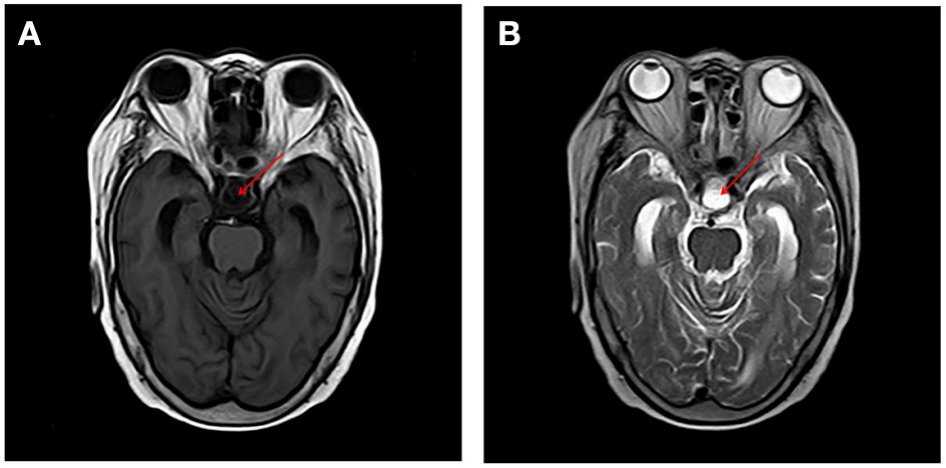
MRI of the brain revealed change after removal of pituitary tumor and no acute cerebral infarction, cerebral hemorrhage, and inflammatory changes were found on T1 and T2 sequences [T1, T1-Weighted Magnetic Resonance Imaging; T2, T2-Weighted Magnetic Resonance Imaging **(A)** T1, **(B)** T2].

**Figure 2:**
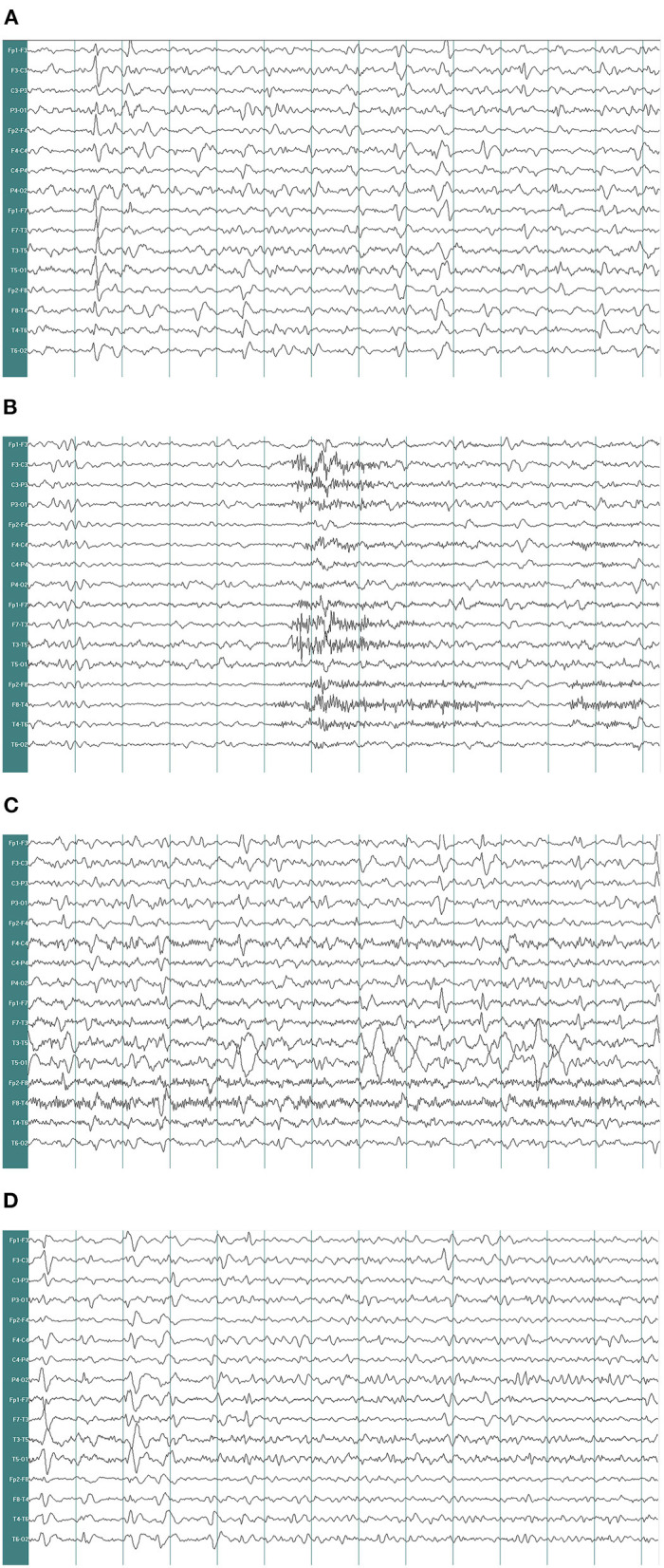
Electroencephalogram recorded. EEG demonstrates δ and θ waves in each lead as background, mingled with sharp waves, spike waves, sharp-slow waves **(A)**. EEG shows a large number of sharp waves, spike waves, sharp-slow waves, spike-slow waves in the bilateral frontal pole, frontal and anterior temporal areas, paroxysmal or continuous, sometimes accompanied by evolution trend **(B–D)** (paper speed: 30 mm/s, high-frequency filter (HF): 30 Hz, low-frequency filter (LF): 0.53 Hz, notchfilers: 50 Hz, sensitivity:7 μv/mm. EEG A provided EEG background and the EEG B showed epileptic discharges).

A 67-year-old woman with diabetes was admitted to the emergency department due to two generalized tonic-clonic seizures and loss of awareness in the interictal period. The patient had never experienced seizures before. Half a year ago she had surgical resection of pituitary tumor and did not get regular hormone replacement treatment. In the days leading up to the seizures, she did not have any signs of discomfort such as fever or headache. In the emergency room diazepam (5 mg, i.v.) and sodium valproate (50 mg, intravenous pumped at the rate of 160 mg/h) were given immediately as antiepileptic therapy, the seizures were terminated in several minutes and the patient fell in state of sedation shortly after, during which the patient could gradually open her eyes, answer simple questions and no more convulsions were observed. Routine blood tests in the emergency department showed hyperglycaemia (13.0 mmol/L), mild hypernatremia (151.7 mmol/L), and hyperchloremia (111.3 mmol/L). Then she was admitted to the neurology department and rescue measures including oxygen inhalation, antiepileptic therapy with sodium valproate (56 mg/h, 1 mg/kg/h), rehydration, maintaining water, and electrolyte balance were continued. About 3 h later, the patient passed into a lethargic state and she could only open her eyes on hearing our call but could not respond in words (GCS score of 8). The lethargic state continued for several hours and the patient fell in coma then (GCS score of 5), which lasted for the following 3 days. During this period the sodium valproate was pumped for 36 h continuously (56 mg/h for 24 h, 50 mg/h for 12 h, 24 mg/h for 12 h, total dose of 2,232 mg) and no remarkable facial or limb spasms or convulsions were recorded from admission. Tests of thyroid function, adrenocorticotropic hormone (ACTH), growth hormone (GH), cortisol, and sex hormone were conducted and the results supported the diagnosis of hypopituitarism (Table 1). MRI of the head was performed and no acute cerebral infarction, cerebral hemorrhage, and inflammatory changes were found (Figure 1). A lumbar puncture was performed to the patient on the 2nd day of admission. The cerebrospinal fluid (CSF) showed elevated protein levels (0.60/L, reference range 0.15–0.45 g/L) and increased chloride (137.8 mmol/L, reference range 118–132 mmol/L) and normal glucose levels and CSF pressure (Table 1). Autoimmune encephalitis and viral encephalitis tests were both negative, and thus the diagnosis of encephalitis was ruled out. A 24-h electroencephalogram (EEG) revealed sharp waves, spike waves, sharp-slow waves, spike-slow waves in the bilateral frontal region on a continuous slow-wave background with a trend of evolution, and the EEG also recorded paroxysmal electrical activities in this region (Figure 2). Combining the symptoms and examinations, the diagnosis of NCSE was considered. On the 3rd day of admission, diazepam (10 mg) was intravenously injected and the patient turned to a state of lethargy gradually and could open her eyes under painful stimulation, confirming the diagnosis of NCSE. Unfortunately, her family refused further treatment and she was discharged on the 3rd day, and she died of respiratory failure induced by epilepsy 5 days after her discharge from our hospital.

**Table 1:** Laboratory data.

**Test**	**Result**	**Normal range** ** of value**	**Test**	**Result**	**Normal range** ** of value**
GH	0.031 ng/ml	(0.126–9.880)	FT3	2.40 pmol/L	(3.28–6.47)
Cortisol (00:00)	18.67 nmol/L	(185–624)	FT4	9.51 pmol/L	(7.64–16.03)
Cortisol (08:00)	17.28 nmol/L	(185–624)	TT3	0.24 nmol/L	(1.01–2.48)
Cortisol (16:00)	25.54 nmol/L	(185–624)	TT4	54.30 nmol/L	(69.97–152.52)
ACTH (00:00)	<1.00 pg/ml	(7.20–63.40)	TSH	0.04 uIU/ml	(0.56–5.91)
ACTH (08:00)	<1.00 pg/ml	(7.20–63.40)	Estradiol	0.02 pg/ml	(10–30)
ACTH (16:00)	<1.00 pg/ml	(7.20–63.40)	Testosterone	<0.1 ng/ml	(0.1–0.75)
Prolactin	28.67 ng/ml	(2.74–19.64)	Progesterone	<0.08 ng/ml	(<1.0 ng/ml)
LH	0.30 mIU/ml	(10.87–58.64)	FSH	10.87 mIU/ml	(16.74–113.5)
CSF protein	0.60 g/L	(0.15–0.45)	CSF chloride	137.8 mmol/L	(118–132)
CSF glucose	3.82 mmol/L	(2.5–4.2)	CSF white blood cell	0.002 ×10^9^/L	(0–8)
CSF red blood cell	0.002 ×10^12^/L	0			

*Tests of hormone levels support the diagnosis of hypopituitarism.*

*GH, growth hormone; ACTH, adrenocorticotropic hormone; LH, luteinising hormone; FSH, folliclestimulating hormone; TSH, thyroid-stimulating hormone; FT3, free triiodothyronine; FT4, free thyroxine; TT3, total triiodothyronine; TT4, total thyroxine*.

## Discussion

NCSE refers to a continuous nonconvulsive seizure that lasts for more than 30 min, or multiple nonconvulsive seizures happening during a period of more than 30 min and between which cognitive, motor, and/or sensory disorders is not fully recovered (4). Patients with NCSE can present as a variety of clinical manifestations, among which altered mental status is the most common (5). As the symptoms of NCSE are various, the diagnosis of NCSE relies on the combination of clinical presentation, electroencephalography findings, and clinical and electrographical reactions to antiepileptic treatments (4). The transition from generalized convulsive status epilepticus (GCSE) to NCSE is common: studies found that NCSE was quite common in patients in the ICU, accounting for about 50% (4). In our case, the patient initially presented as GCSE and went into coma which was diagnosed as NCSE finally.

The aetiology and risk factors of NCSE are complex, including acute and chronic pathological conditions (4). The role of endocrine system diseases in the pathogenesis and treatment of epilepsy has been studied continuously.

Hypopituitarism is defined as deficiency of one or more pituitary hormones produced in the anterior lobe and posterior lobe, including GH, the gonadotropins follicle stimulating hormone (FSH) and luteinizing hormone (LH), ACTH, thyroid stimulating hormone (TSH), prolactin, oxytocin, and antidiuretic hormone (ADH) (6).

Metabolic encephalopathy can also present with disorders of consciousness and it can be differentiated from NCSE by etiology, laboratory examination, and EEG. While increasing slowing of the waking background frequency was observed in hyperglycemia and hypoglycemia, hyponatremia may initially produce posterior slowing followed by more diffuse delta activity (7), hypopituitarism and hypoadrenalism may cause diffuse theta and delta activity. Furthermore, diffuse suppression with scant activity may be found in patients with hypothyroidism (8).

The relation between hormones and epilepsy is complicated. Concerning sex hormones, while androgens, progesterone and its metabolites have been found to have anticonvulsant effects, proconvulsant effects of estrogens were well-described (9). Studies have found that changes in hormone levels correspond to changes in seizures' frequency, which was considered as the effect of hormones on brain excitability (9), and this association was well-documented in catamenial epilepsy, in which the distribution of seizure numbers varied across the days of the menstrual cycle (10). Our patient showed remarkably low level of sex hormones including estradiol, FSH, LH, progesterone, and testosterone, making the evaluation of the role of any single hormone in the seizures complicated. Studies have found increased serum prolactin in about 67% of cases after complex-partial seizures or generalized tonic-clonic seizures (11, 12). Our patient had a relatively high level of prolactin, which was consistent with the previous reports.

The use of sex steroid hormones or their analogs for the treatment of epilepsy has been studied (13). It has been shown that supplementation of progesterone was effective in the management of seizure in patients with anovulatory cycles (14). Indeed, studies have confirmed that hormones including corticosteroids and ACTH were effective in the management of pediatric epilepsies including West syndrome, the Landau–Kleffner syndrome, other epilepsies, and epilepsy syndromes (15, 16).

It has been reported increasingly that stress plays a role in precipitating seizures (17, 18). Preclinical models of epilepsy showed that neuronal excitability and seizure susceptibility were both influenced by stress hormones (19). The hypothalamic-pituitary-adrenal axis is the main neuroendocrine system activated by stress (20). Studies on animal models revealed that neuronal excitability and seizure threshold were affected by stress hormones including corticotrophic hormone and corticosterone (21). It was reported that excitability of neurons was increased under the influence of corticotropin-releasing hormone (CRH) both *in vivo* and *in vitro*, and thus seizure activity was induced (22). Given the negative feedback effect of the hypothalamus-pituitary-adrenal axis, our patient should theoretically have high levels of CRH which may induce seizures.

Under basal conditions, stress hormones can also affect disease activity in seizures (23). In most of the studies, it was shown that increases in cortisol levels were a promoter during epileptogenesis (24, 25). But Ostrowska's team had the opposite conclusion and they found that patients with epilepsy had lower cortisol levels than those without epilepsy (26). Pritchard et al. (27) found that before generalized tonic-clonic seizures and complex partial seizures, the cortisol levels increased, while before simple partial seizures and secondary generalized seizures, the results were opposite (28). More importantly, patients with epilepsy often showed increased cortisol levels (29, 30). While the mechanisms of interaction between stress and seizures are not entirely clear, both of the two would have impact on cortisol levels.

Zhang and Liu (28) conducted a study analysing ACTH and cortisol levels during sleep seizures had found that during and after an epileptic seizure, the ACTH and cortisol levels were higher than those before a seizure. The researchers believed that epileptic seizures may be induced by decrease in ACTH and cortisol levels. A possible explanation may be that at night the decrease in ACTH levels triggers the release of CRH through negative feedback regulation, and the release of CRH increase epilepsy susceptibility and lower epilepsy threshold (21, 31).

Although the mechanisms of epileptogenesis still remain to be unclear, it is well-proved that mitochondrial dysfunction (32, 33) and oxidative stress (34) play important roles in this process. Molecular evidences have revealed that thyroid hormones are involved in normal mitochondrial biogenesis and function (35) and decreased activity of thyroid hormones is related to mitochondrial dysfunction (36). As to oxidative stress, both hyperthyroidism and hypothyroidism can affect antioxidant/oxidant balance (37). The impact of thyroid hormones in different aspects of epilepsy has been demonstrated (37). Thyroid function tests showed obvious hypothyroidism in our patient, which may also play an important role in the occurrence of epilepsy.

Treatments of NCSE should be based on expert opinions as well as individual aetiology including critical illness, subtherapeutic AED levels, etc. Recommendations for first-line treatment of NCSE are usually benzodiazepines (4). In our patient, recovery of impaired consciousness after the use of diazepam confirmed the diagnosis of NCSE. Regrettably, the patient did not receive systemic therapy and passed away finally.

## Conclusion

In summary, our case, an old woman with hypopituitarism presenting with NCSE, led us to further explore the complex relationship between epilepsy and hormones, which contributes to the accurate diagnosis and treatment.

## Data Availability Statement

The original contributions presented in the study are included in the article/supplementary material, further inquiries can be directed to the corresponding author/s.

## Ethics Statement

The study involving a human participant was reviewed and approved by the Ethics committee of Jincheng People's Hospital, Jincheng, China. The patient provided her written informed consent to participate in this study. Written informed consent was obtained from the individual for the publication of any potentially identifiable images or data included in this article.

## Author Contributions

LJ put forward research ideas. LX took the responsibility of communicating with the patient's family and obtaining the authorization in this paper. HL was responsible for drafting articles. FY revised the article. HC and WL were responsible for literature searches and final proofreading. All authors contributed to the article and approved the submitted version.

## Conflict of Interest

The authors declare that the research was conducted in the absence of any commercial or financial relationships that could be construed as a potential conflict of interest.

## Publisher's Note

All claims expressed in this article are solely those of the authors and do not necessarily represent those of their affiliated organizations, or those of the publisher, the editors and the reviewers. Any product that may be evaluated in this article, or claim that may be made by its manufacturer, is not guaranteed or endorsed by the publisher.
